# Barriers to the recognition and evaluation of abnormal uterine bleeding among individuals at increased risk for endometrial Cancer

**DOI:** 10.1016/j.gore.2026.102214

**Published:** 2026-09-24

**Authors:** Shariska P. Harrington, Negar Taheri, Manisha Salinas, Andilyn Mehltretter, Nicholas McConnell, Martha O'Neill, Arushi Agarwal, Donna Hochberg, Tomasz Beer, Mark Camardo, Elle Kielar-Grevstad, Jamie N. Bakkum-Gamez

**Affiliations:** a Department of Obstetrics and Gynecology, Division of Gynecologic Oncology, Mayo Clinic, Rochester, MN, USA.; b Center for Health Equity and Community Engaged Research, Mayo Clinic, Jacksonville, FL, USA.; c Health Advances, 275 Grove Street 1E-Suite 310, Newton, MA 02466, USA; d Exact Sciences, 5505 Endeavor Lane, Madison, WI 53719, USA

**Keywords:** Endometrial cancer, Abnormal uterine bleeding, Survey, Diagnostic evaluation

## Abstract

**Objective:**

To evaluate knowledge of abnormal uterine bleeding (AUB) and postmenopausal bleeding (PMB) as symptoms of endometrial cancer (EC), assess understanding of diagnostic evaluation, and identify barriers and facilitators to care.

**Methods:**

We conducted a web-based survey of participants (*N* = 603) with a history of AUB/PMB and increased risk for EC. The survey, informed by semi-structured interviews, assessed symptom recognition, knowledge of EC, familiarity with diagnostic evaluation, and healthcare-seeking behaviors. Descriptive statistics, chi-square tests, and multivariable logistic regression evaluated associations between demographic, clinical factors and study outcomes.

**Results:**

Although 78% recognized abnormal bleeding within six months, 21% reported delayed recognition and 25% had not sought evaluation. Only 12% identified AUB/PMB as a potential symptom of cancer, with participants aged ≥45 years less likely to make this association. While 75% sought medical care, 28% reported that their healthcare provider was not concerned about their bleeding; among these participants, 60% reported experiencing symptom dismissal. Familiarity with endometrial biopsy was lower among Medicaid-insured participants (OR 0.50, 95% CI 0.33–0.77). More convenient testing options increased self-reported motivation to seek evaluation.

**Conclusions:**

Knowledge gaps, delayed symptom recognition, and experiences of symptom dismissal identify potential barriers along the pathway to evaluation of abnormal bleeding.

## Highlights

• AUB/PMB is poorly recognized as a symptom of endometrial cancer.
• Delayed recognition of abnormal bleeding is common among women at risk for EC.
• Knowledge of diagnostic evaluation, including endometrial biopsy, is limited.
• Barriers to AUB evaluation include low concern, access, and fear-related factors.
• Convenient testing options increase motivation to seek evaluation especially among Black women.

## 1 Introduction

Abnormal uterine bleeding (AUB) is common and has numerous benign etiologies; however, it is also the most common presenting symptom of endometrial cancer (EC) (Munro et al., 2018; Committee on Practice, B.-G, 2012). The significance of abnormal bleeding and recommended evaluation differ across the menopausal transition. In premenopausal individuals, the need for endometrial sampling is guided by age, persistence of bleeding, response to medical management and EC risk factors (Committee on Practice, B.-G, 2012). In contrast, postmenopausal bleeding (PMB) warrants prompt evaluation because of its established association with EC (Clarke et al., 2018), with current recommendations incorporating transvaginal ultrasonography and endometrial tissue sampling in the initial evaluation (American College of, O. and Gynecologists, 2026). Timely evaluation therefore depends on patients recognizing when bleeding is abnormal, seeking medical care, and receiving appropriate diagnostic evaluation.

Prior studies suggest vulnerabilities at each of these steps. Qualitative studies of AUB conducted outside the United States have described normalization of abnormal bleeding and uncertainty regarding which bleeding patterns warrant medical evaluation (Henry et al., 2020a; Henry et al., 2020b; Ibrahim et al., 2024). In the United States, Doll et al. found that Black women with EC were less likely than White women to have documented PMB followed by guideline-concordant evaluation, and that nonguideline-concordant diagnostic pathways were associated with advanced-stage disease (Doll et al., 2018). Subsequent qualitative work identified menopause and EC knowledge, prior healthcare experiences, reproductive conditions including fibroids, and healthcare-provider response as potential contributors to care delay among women with EC (Doll et al., 2022).More recently, only 23% of EC survivors who experienced PMB reported prediagnosis awareness that bleeding could be a symptom of EC (Wise et al., 2026).

Provider-level factors may further influence the diagnostic pathway. In a national survey of first-line providers, knowledge gaps regarding EC and its risk factors were common, and nonguideline-concordant evaluation was reported in 14%–41% of clinical scenarios (Sage et al., 2023).More recent qualitative interviews with physicians across multiple specialties similarly identified uncertainty regarding EC diagnostic guidelines and racial disparities in EC diagnosis (Gross et al., 2025).Together, these studies suggest that delays in evaluation may reflect both patient- and healthcare-level factors.

However, U.S. studies have largely examined healthcare providers or individuals already diagnosed with EC, while qualitative studies examining experiences with AUB more broadly have predominantly been conducted outside the United States. Less is known about how a diverse U.S. population experiencing AUB or PMB recognizes its own bleeding, understands its potential significance and diagnostic evaluation, and makes decisions about seeking care. Importantly, studies of patients with EC cannot characterize individuals with abnormal bleeding who have not yet sought medical evaluation.

Therefore, we conducted a survey of a racially and ethnically diverse U.S. based population with a history of AUB or PMB to evaluate recognition of abnormal bleeding, knowledge of its association with EC, familiarity with diagnostic evaluation, and barriers and facilitators to seeking care. We also evaluated demographic and clinical factors associated with symptom recognition, diagnostic knowledge, and care-seeking intentions.

## 2 Methods

### 2.1 Survey Design

To quantify knowledge of AUB/PMB as a potential symptom of EC and identify barriers and facilitators to evaluation for AUB/PMB, we conducted an online quantitative survey of participants at risk for EC. The survey instrument was developed specifically for this study and was informed by semi-structured qualitative interviews with 16 individuals who had experienced AUB or PMB. Interviews explored participants' experiences with abnormal bleeding, symptom recognition, barriers to seeking evaluation, experiences with diagnostic evaluation, and factors that might facilitate care-seeking. Feedback was aggregated across interviews to identify recurring patient experiences relevant to the study objectives and was used to inform survey content and item wording. The interviews were conducted to inform survey development rather than to generate a formal qualitative thematic model; therefore, thematic saturation was not formally assessed. The resulting survey was pilot tested with two participants from the qualitative interviews prior to administration.

The survey consisted of 104 items, including 22 screening eligibility questions and 82 survey questions. The instrument was originally developed in collaboration with Exact Sciences for market research purposes. Of the 82 survey questions, 64 assessed experiences related to AUB/PMB, whereas 18 were specific to the product under evaluation and were excluded from the current analysis. Based on concepts identified during the formative qualitative interviews, participants were also asked whether greater convenience of diagnostic testing, including the hypothetical availability of a self-administered testing option, would increase their motivation to seek evaluation for abnormal bleeding. These questions did not refer to a specific commercially available test. Questions related to the product under evaluation were positioned at the end of the survey and were excluded from this study.

The survey utilized adaptive questioning (skip logic), whereby respondents were directed to follow-up questions or advanced to the next relevant item based on their responses. This approach minimized respondent burden while ensuring the collection of pertinent information. Questions were presented one at a time and required a response before participants could proceed to the next item (forced completion). Respondents were not permitted to return to previously completed questions. Because respondents completed survey items sequentially without the ability to revisit prior questions, we do not anticipate that product-related questions at the end of the survey influenced responses to earlier AUB-related items.

Health Advances (Boston, MA) was responsible for survey design, programming, administration, and data collection. The survey instrument used for this study, including the 22 screening questions and 64 AUB-related questions, is provided in Supplementary data 1.

To ensure high-quality research, the survey respondents were recruited to take an online survey by a third-party market research vendor (Dynata) in a double-blinded fashion so that the identity of the respondent was not revealed to us (the sponsor), and the sponsor of the study was not revealed to the respondent in compliance with industry standards. Dynata, a commercial survey research company, maintains a large U.S.-based online research panel. The panel includes individuals from the general population who have opted to participate in survey research. The survey respondents were recruited from April to May 2024. Dynata shared a link to the web-based online survey with its general population panel, inviting panel members to participate in the research. If participants met the screening criteria (below), they proceeded to complete the 82-item survey after providing consent. All respondents who completed the survey were paid an honorarium. This survey research project was exempt from IRB oversight in accordance with the Department of Health and Human Services regulations 45 CFR 46.104(d) (2), with IRB review and final approval achieved on the Advarra CIRBI Platform, IRB number 00078362.

### 2.2 Survey participant eligibility criteria

Survey respondents were first asked a set of screening questions to assess their eligibility and those who met screening criteria were invited to participate. Specifically, for eligibility all participants were required to have experience with abnormal bleeding. Abnormal bleeding was defined as any of the following: vaginal bleeding between periods (including any bleeding between periods, such as spotting, light pink or brown blood, and bleeding after intercourse), heavy vaginal bleeding (needing to change a tampon/pad every 1–2 h or bleeding lasting longer than 8.

days), vaginal bleeding after menopause (including any bleeding after menopause such as spotting, light pink or brown blood, and bleeding after intercourse), and/or red, brown, or pink colored vaginal discharge. Additional eligibility criteria are as follows: all participants had to be assigned female sex at birth, have a high school diploma or higher level of education, and if between the age of 30–45 years old had to have at least one risk factor of EC including obesity (BMI ≥30 kg/m2), a family history of gynecological cancers, polycystic ovary syndrome, unopposed estrogen replacement therapy, or tamoxifen use. Exclusion criteria included current residence outside of the United States, residence in Washington State (due to My Health My Data Act), age < 30 years old and those with less than a high school diploma or GED.

Demographic information was obtained, including self-reported race, ethnicity, age, household income, geographic location, employment status, education level, and insurance status. Clinical factors including self-reported menopausal status, diagnosis of PCOS, obesity, family history of cancer, use of hormone replacement therapy and tamoxifen were also obtained.

Sampling targets were used to ensure representation of racial/ethnic groups (goal set at a minimum of 150 participants/group), geographic locations, household income levels, levels of educational attainment, insurance payors and menopausal status (Supplementary Data 1). To achieve target enrollment, demographic quota parameters were modified during survey recruitment in response to challenges recruiting certain population subgroups. Rural residence quotas were adjusted from >20% to >15% of the final sample. Race/ethnicity quotas were revised from equal 20% targets across five groups to target ranges of 20–22% for Black/African American, Asian, Hispanic, and White/Caucasian respondents, and 15–20% for respondents identifying as other races/ethnicities. Menopausal status quotas were also modified, increasing the premenopausal target from <20% to <30%, setting the perimenopausal target at 50%, and maintaining the postmenopausal target at <60%. These modifications affected recruitment quotas only and did not alter survey content, eligibility criteria, study procedures, or respondent-facing materials. These quota modifications were implemented prospectively during recruitment to improve feasibility while maintaining demographic diversity in the study population.

### 2.3 Statistical analysis

We used descriptive statistics to summarize the survey results in the overall sample and stratified by key demographics including race, ethnicity, age, menopausal status, household income, geographic location, education, and insurance status. Differences in responses were tested via chi-square analysis, and multivariate logistic regression models were used to adjust for potential confounding. Clinical variables were selected a priori based on clinical relevance and each model had different prespecified covariates relevant to the outcome being evaluated. A *p*-value of <0.05 indicated a statistically significant association. There was no adjustment made for multiplicity as all results are hypothesis generating. All analyses were conducted using SAS EG version 7.1.

## 3 Results

A total of 11,166 individuals initiated the survey of whom 10,261 did not meet eligibility criteria. Among the 905 participants who met eligibility criteria and initiated the survey, 750 completed the survey and passed data-quality review, corresponding to an analytic retention rate of 82.9%. Because recruitment occurred through an opt-in online panel and the total number of panel members invited or exposed to the survey invitation was unavailable, a conventional survey response rate could not be calculated.

Participants with a prior diagnosis of endometrial cancer (*n* = 75) or history of uterine surgery, including hysterectomy (*n* = 72) were excluded, resulting in a final analytic cohort of 603 participants.

### 3.1 Participant demographics

Most participants were aged 45–70 years (74.3%), had completed at least some college education (63.4%), were employed (66.3%), and lived in an urban or suburban area (83.7%). The population was racially and ethnically diverse, and 55.4% had private insurance (Table 1). Overall, 172 participants reported PMB and 431 reported bleeding without PMB. Demographics are summarized in Table 1.

**Table 1:** **Participant characteristics by time to recognition of abnormal uterine bleeding (AUB).** Characteristics of survey respondents (*N* = 603) are presented overall and stratified by time to recognition of AUB (1–6 months vs >6 months). Values are shown as number (percentage). *P* values were calculated to compare differences between groups.

Characteristic	Total (*N* = 603)	Time to Recognize AUB		*p*-value
		1–6 months (*N* = 474)	Greater than 6 months (*N* = 129)	
Age, n (%)				
30–39 years	86 (14.26%)	67 (14.1%)	19 (14.7%)	0.0967
40–44 years	38 (6.30%)	27 (5.7%)	11 (8.5%)	
45–51 years	180 (29.85%)	153 (32.3%)	27 (20.9%)	
52–59 years	164 (27.20%)	121 (25.5%)	43 (33.3%)	
60–70 years	104 (17.25%)	84 (17.7%)	20 (15.5%)	
71–79 years	31 (5.14%)	22 (4.6%)	9 (7.0%)	
				
Race, n (%)				
White	132 (21.89%)	91 (19.2%)	41 (31.8%)	0.0003
Black	136 (22.55%)	107 (22.6%)	29 (22.5%)	
Asian	104 (17.25%)	75 (15.8%)	29 (22.5%)	
Hispanic	130 (21.56%)	109 (23.0%)	21 (16.3%)	
Other	101 (16.75%)	92 (19.4%)	9 (7.0%)	
				
Education, n (%)				
Graduate	92 (15.26%)	72 (15.2%)	20 (15.5%)	0.5256
Bachelors	178 (29.52%)	141 (29.7%)	37 (28.7%)	
Associates	112 (18.57%)	93 (19.6%)	19 (14.7%)	
Diploma	221 (36.65%)	168 (35.4%)	53 (41.1%)	
				
Employment Status, n (%)				
Working	400 (66.33%)	312 (65.8%)	88 (68.2%)	0.6099
Not Working	203 (33.67%)	162 (34.2%)	41 (31.8%)	
				
Urban/Rural Classification, n (%)				
Urban	273 (45.27%)	223 (47.0%)	50 (38.8%)	0.1039
Suburban	232 (38.47%)	172 (36.3%)	60 (46.5%)	
Rural	98 (16.25%)	79 (16.7%)	19 (14.7%)	
				
Household Income, n (%)				
Less than $25,000	82 (13.60%)	61 (12.9%)	21 (16.3%)	0.5447
$25,001-75,000	208 (34.49%)	161 (34.0%)	47 (36.4%)	
$75,001-125,000	142 (23.55%)	112 (23.6%)	30 (23.3%)	
Greater than $125,000	171 (28.36%)	140 (29.5%)	31 (24.0%)	
				
Payor Status, n (%)				
Medicaid	139 (23.05%)	116 (24.5%)	23 (17.8%)	0.0508
Medicare/MedAdv/Veterans	98 (16.25%)	80 (16.9%)	18 (14.0%)	
Private Insurance	334 (55.39%)	258 (54.4%)	76 (58.9%)	
Uninsured/Self-Insured	32 (5.31%)	20 (4.2%)	12 (9.3%)	

### 3.2 Recognition of abnormal bleeding

Most participants (474/603, 78.6%) recognized their bleeding as abnormal within 1–6 months of symptom onset, whereas 129 (21.4%) reported taking longer than 6 months to recognize their bleeding as abnormal. The distribution of race and ethnicity differed between participants with and without delayed recognition (*p* < 0.001), while differences by insurance status approached statistical significance (*p* = 0.05). Age, education, employment status, geographic setting, and household income were not associated with delayed recognition.

Recognition differed by PMB status. Participants with PMB more frequently recognized their bleeding as abnormal within 1–6 months compared with participants without PMB (85.5% vs 75.9%), whereas delayed recognition beyond 6 months was less frequent (14.5% vs 24.1%; *p* = 0.011) (Supplementary Data 2).

More than 1 year elapsed before recognition of abnormal bleeding among 21% of postmenopausal participants, 23% of participants with lower household income, and 20% of participants with no education beyond high school. In a multivariable model adjusted for race and ethnicity and whether a healthcare provider inquired about bleeding before the participant disclosed the symptom, premenopausal participants had higher odds of delayed recognition (>6 months) compared with perimenopausal participants (OR 1.90, 95% CI 1.02–3.42).

### 3.3 Knowledge of abnormal bleeding as a potential cancer symptom

More than one in four (162/603) participants indicated they were not concerned about their abnormal bleeding and reported that it was not something to worry about. Among these participants, 37% believed that it would go away on its own.

Among the 441 participants who reported concern about their bleeding, 53 (12.0%) identified abnormal bleeding as a potential symptom of cancer, and 20 of these 53 participants (37.7%) specifically identified endometrial cancer. Knowledge of abnormal bleeding as a symptom of cancer differed by age. After adjusting for education level, payor status, receipt of diagnostic evaluation, and history of postmenopausal bleeding, participants younger than 45 years had higher odds of recognizing abnormal bleeding as a symptom of cancer compared with participants aged 45 years and older (OR 2.20, 95% CI 1.07–4.46) (Fig. 1a).

**Fig. 1:**
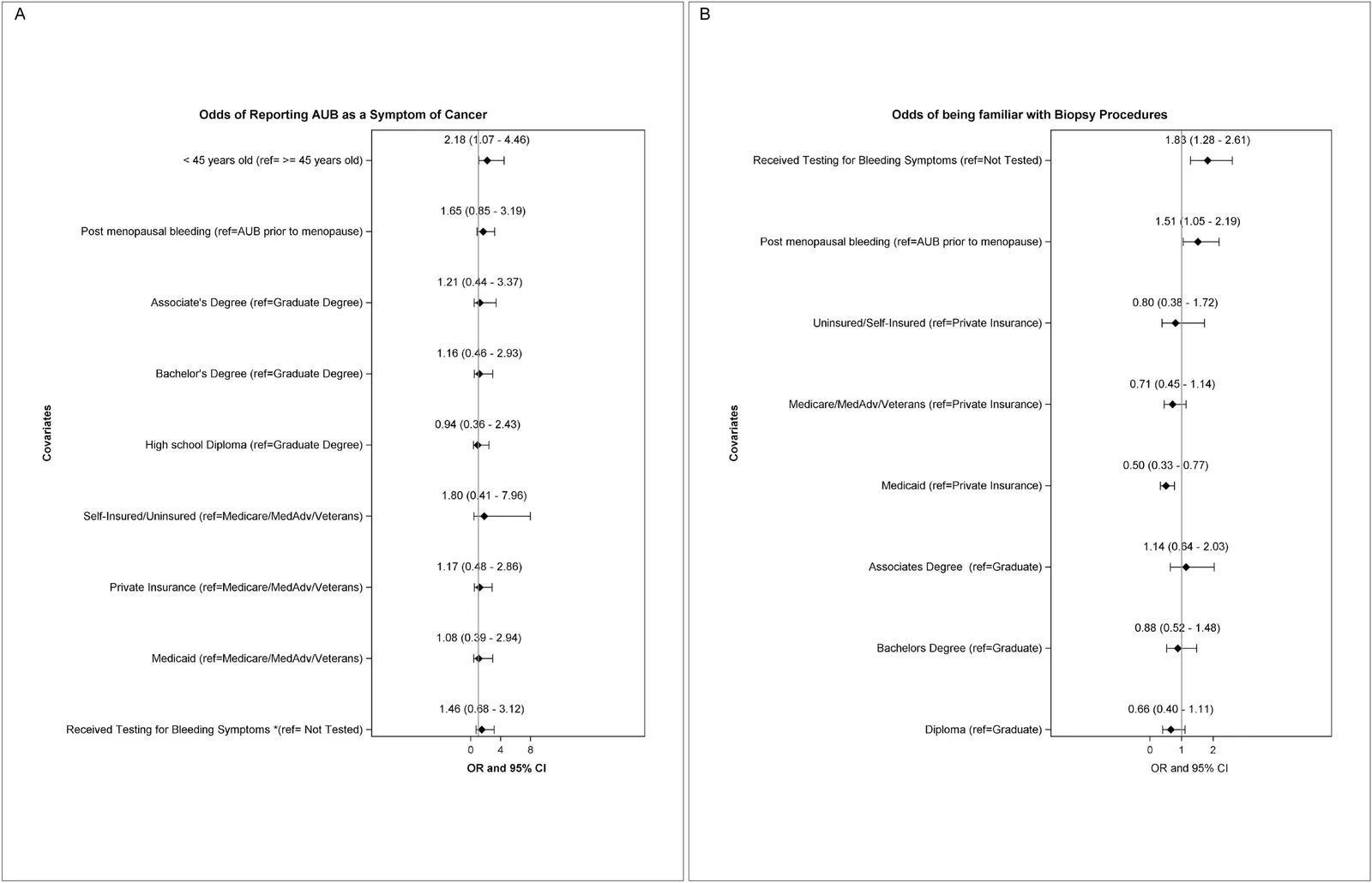
**Factors associated with recognition of abnormal uterine bleeding (AUB) as a symptom of cancer and familiarity with endometrial biopsy procedures in all participants (*n*** **=** **603).** (A) Adjusted odds ratios (ORs) and 95% confidence intervals (CIs) for reporting AUB as a symptom of cancer. (B) Adjusted ORs and 95% CIs for familiarity with endometrial biopsy procedures. Models include relevant demographic and clinical covariates. ORs >1 indicate higher odds of the outcome. Error bars represent 95% CIs.

Among the 20 participants who reported knowing AUB/PMB was a symptom of EC, 11 (55%) reported learning this from conversations with their doctor. Because respondents could select more than one source of information, friends and family members and online searches were also frequently cited, reported by 65% (13/20) of participants. Across the full study population, 68% ranked a healthcare provider as their preferred source of information for learning about causes of abnormal bleeding and endometrial cancer risk (Fig. 2).

**Fig. 2:**
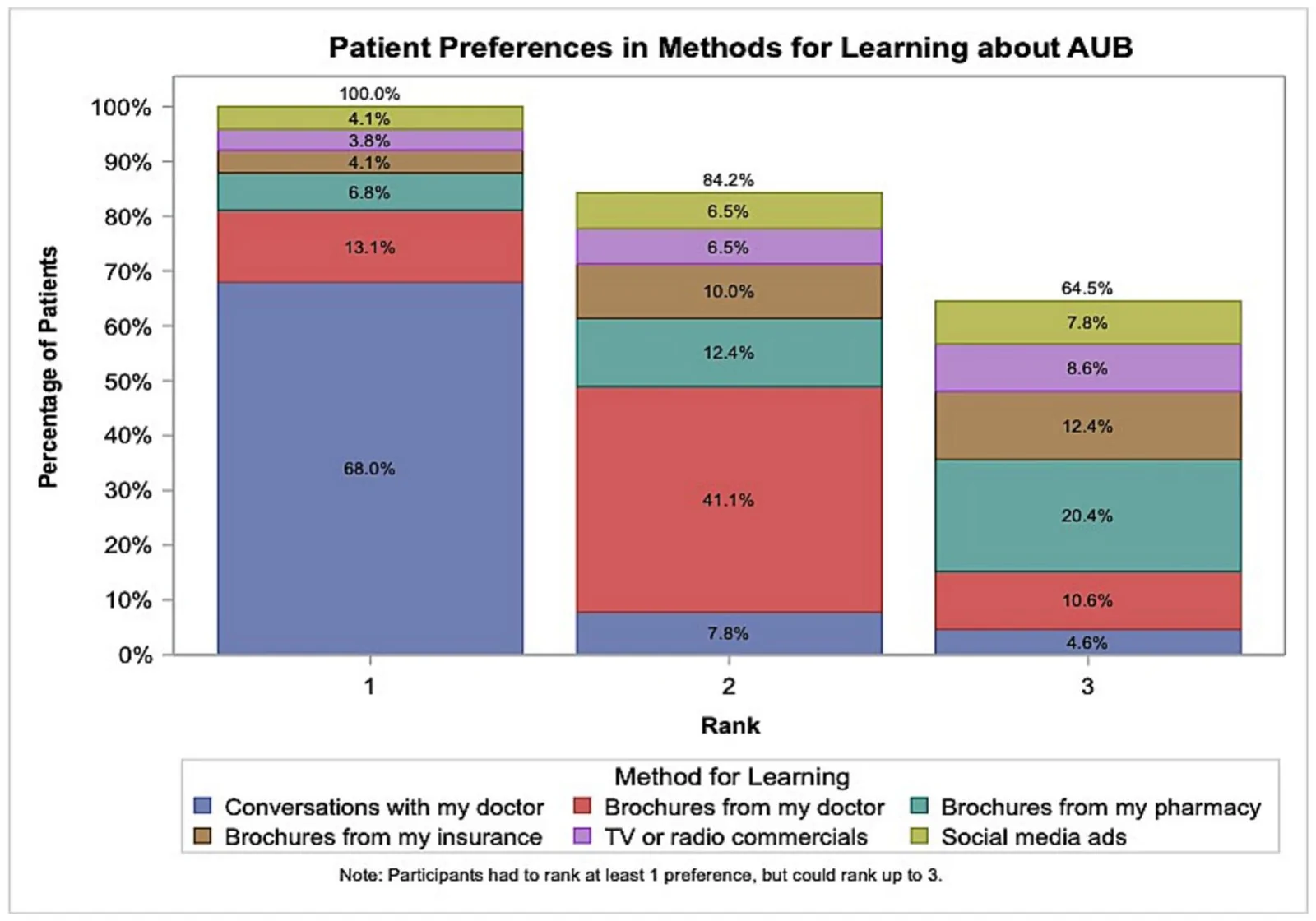
**Ranked participant preferences for methods of learning about AUB.** Participants ranked their preferred methods for receiving information about AUB etiology. Stacked bars represent the percentage of respondents selecting each method at each rank (1 = highest preference). Total percentages for each rank reflect the proportion of participants who provided that rank (rank 1: 100%, rank 2: 84.0%, rank 3: 65.2%). Participants could rank up to three options but were required to select at least one preference.

### 3.4 *Diagnostic evaluation and familiarity with diagnostic testing*

Overall, 450 participants (74.6%) reported seeking medical care for abnormal bleeding, and 387 (64.2%) reported undergoing diagnostic evaluation. Gynecologists were the most frequently reported provider type (262/450, 58.2%), followed by primary care providers (121/450, 26.9%) and urgent care/walk-in clinics (21/450, 4.7%); all other provider types were reported by fewer than 4% of participants (Supplementary data 3). In exploratory analyses, the proportion of participants who sought care was nearly identical among those with and without PMB (74.4% vs 74.7%; *p* = 1.00).

Participants were also surveyed about familiarity with diagnostic tests to evaluate AUB/PMB. Among the 603 participants who identified tests they were familiar with, 396 (65.7%) were familiar with pelvic exam, 257 (42.6%) were familiar with transvaginal ultrasounds and 282 (46.8%) were familiar with endometrial sampling, including endometrial biopsy and D&C. In multivariable logistic regression that adjusted for payor status, education level and receiving diagnostic evaluation for bleeding symptoms, participants who experienced postmenopausal bleeding had higher odds of being familiar with biopsy procedures compared to those who had bleeding before menopause (OR 1.51, 95% CI 1.05–2.19). Participants insured by Medicaid had lower odds of being familiar with biopsy procedures compared to those who had private insurance (OR 0.50, 95% CI 0.33–0.77) (Fig. 1b).

### 3.5 Barriers to diagnostic evaluation

One in four participants (125/450) reported that their health care provider was not concerned about their bleeding symptoms, including 25.6% of gynecology encounters and 36.4% of primary care encounters (Supplementary Data 3). Among those 125 participants, 60% (75/125) reported feeling dismissed. Participants reporting PMB were less likely to report that a provider was not concerned about their bleeding than those with AUB (15.1% vs 23.0%; *p* = 0.035) (Supplementary Data 2). Those who experienced dismissal subsequently sought information from friends or family, conducted their own research, or pursued a second opinion from another physician.

At the time of the survey, 153 (25.4%) participants reported that they had not yet sought care for AUB/PMB. The top reasons participants reported for not seeing a health care provider included believing bleeding would go away on its own (17.6%), not having a PCP or GYN provider (10.5%), believing it would resolve with lifestyle changes (9.2%), believing it was not something to worry about (9.8%), being afraid of bad results (7.2%), not being able to take time away (7.2%), inability to get an appointment (6.5%) or being afraid of a pelvic exam (3.3%) (Table 2).

**Table 2:** **First-ranked reasons for not seeking care for AUB.** Participants (*N* = 153) identified their top reason for not seeking medical evaluation. Values are presented as number (percentage) of respondents selecting each reason as their highest-ranked choice. Participants were required to rank at least one preference but could rank up to three.

Top reason each participant did not seek care	N (%) (*N* = 153)
Go away on its own	27 (17.6%)
I do not have PCP or GYN	16 (10.5%)
AUB not something to worry about	15 (9.8%)
Go away with lifestyle changes	14 (9.2%)
Cannot take time away	11 (7.2%)
Afraid of bad results	11 (7.2%)
Can't get Appointment	10 (6.5%)
Other	10 (6.5%)
Uncomfortable receiving care because of doctor gender	7 (4.6%)
Afraid insurance won't cover it	6 (3.9%)
I do not trust HCP	5 (3.3%)
Afraid of pelvic exam	5 (3.3%)
Afraid of getting treatment	5 (3.3%)
Don't want to pay	4 (2.6%)
Cannot travel	3 (2.0%)
I am afraid of HCP	3 (2.0%)
Afraid of other tests	1 (0.7%)

Participants who did not seek care for their abnormal bleeding were asked about the likelihood of seeing a healthcare provider in the future to evaluate their abnormal bleeding. After adjusting for employment status, payor status, race, and menopausal status, participants with Medicare/MedicareAdv/Veterans insurance had higher odds of responding ‘more likely’ to seeing a doctor in the future compared to participants covered by Medicaid (OR 5.31, 95% CI 1.15–24.5). Additionally, participants covered by private insurance had higher odds of responding more likely’ to seeing a doctor in the future compared to participants covered by Medicaid (OR 3.63, 95% CI 1.00–13.2). Premenopausal participants had lower odds to respond ‘more likely’ to seeing a doctor in the future compared to perimenopausal participants (OR 0.31, 95% CI 0.11–0.86). Postmenopausal participants had lower odds of responding ‘more likely’ to seeing a doctor in the future compared to perimenopausal participants (OR 0.10, 95% CI 0.04–0.30) (Fig. 3a).

**Fig. 3:**
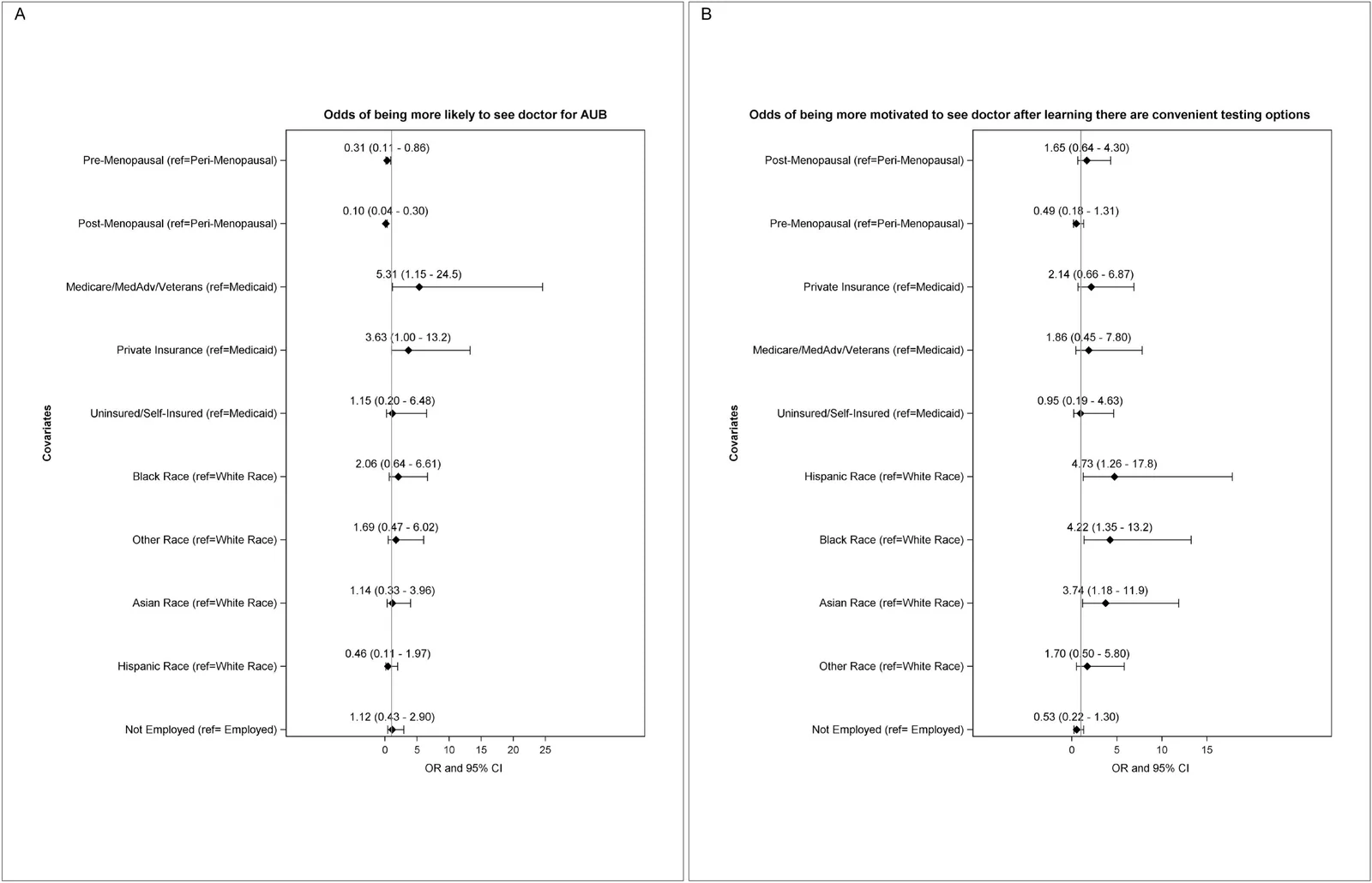
**Factors associated with care-seeking intentions and motivation for evaluation of AUB among those who did not seek care (*n*** **=** **153).** (A) Adjusted odds ratios (ORs) and 95% confidence intervals (CIs) for odds of seeing a doctor for AUB in the future. (B) Adjusted ORs and 95% CIs for being motivated to seek evaluation by convenient testing options. Models include relevant demographic and clinical covariates. ORs >1 indicate higher odds of the outcome; error bars represent 95% CIs.

### 3.6 *Motivation for diagnostic evaluation*

For the 153 respondents who had not sought medical evaluation for their abnormal vaginal bleeding, learning that abnormal bleeding could represent a serious health problem was the most frequently endorsed motivator for seeking care, with 56.2% rating this as very or extremely motivating. Increased bleeding volume (50.3%), more frequent bleeding episodes (52.9%), and the development of additional reproductive symptoms such as cramping (52.3%) were also commonly rated as very or extremely motivating. The ability to obtain an appointment with a physician was rated as very or extremely motivating by 46.4% of respondents.

Greater convenience of testing was very or extremely motivating for 46.4% of participants, and 50.3% reported similar motivation in response to the hypothetical availability of a self-administered testing option. These questions assessed hypothetical facilitators for evaluation and did not refer to a specific commercially available test. After adjustment for menopausal status, employment, insurance coverage, and race and ethnicity, Black (OR 4.22, 95% CI 1.35–13.2), Hispanic (OR 4.73, 95% CI 1.26–17.8), and Asian participants (OR 3.74, 95% CI 1.18–11.9) had greater odds of reporting motivation to seek care in response to more convenient testing compared with White participants. No participant characteristics were significantly associated with motivation after learning that a hypothetical self-administered testing option was available.

## 4 Discussion

In this cross-sectional survey of individuals with abnormal uterine bleeding (AUB) or postmenopausal bleeding (PMB), we identified potential barriers at multiple stages of the pathway from symptom onset to diagnostic evaluation. One in five reported delayed recognition of abnormal bleeding, knowledge of abnormal bleeding as a potential symptom of cancer was limited, fewer than half of participants were familiar with endometrial sampling procedures, and approximately one-quarter had not sought medical evaluation. Among those who did seek care, reports of lack of provider concern and perceived symptom dismissal by healthcare providers were common. Collectively, these findings suggest that barriers to evaluation occur at multiple stages, from recognizing bleeding as abnormal, to understanding its significance, to navigating diagnostic evaluation once care is sought.

Recognition of abnormal bleeding and understanding when abnormal bleeding should be evaluated represents distinct challenges. Premenopausal participants were more likely to report delayed recognition than perimenopausal participants. Prior qualitative studies have similarly described normalization of abnormal bleeding and uncertainty regarding which bleeding patterns warrant medical evaluation (Ibrahim et al., 2024). Participants with PMB recognized their bleeding as abnormal earlier than those without PMB; however, earlier recognition did not translate into greater care-seeking, which was nearly identical among participants with and without PMB. These findings suggest that educational interventions should combine menopausal status-specific guidance regarding abnormal bleeding with information about when medical evaluation is warranted.

Knowledge of the relationship between abnormal bleeding and cancer was also limited. Prior studies have demonstrated incomplete public awareness of endometrial cancer symptoms, although awareness has been higher in predominantly White and highly educated populations (Soliman et al., 2008; Wise et al., 2024). Wise et al. reported that 63% identified PMB as a symptom of endometrial cancer, but fewer than half of postmenopausal participants had received prior provider counseling about PMB (Wise et al., 2024). In our study, healthcare providers were the preferred source of information, highlighting an opportunity for counseling that helps patients both recognize abnormal bleeding and understand when evaluation is warranted. Studies identifying knowledge gaps regarding EC symptoms and risk factors among first-line providers have called for targeted educational interventions (Sage et al., 2023; Gross et al., 2025). Clinician communication may therefore represent an important opportunity to improve timely evaluation of abnormal bleeding.

Healthcare interactions and knowledge of diagnostic evaluation represent additional potential barriers. More than one-quarter of participants who sought care reported that their healthcare provider was not concerned about their bleeding, and 60% of these participants reported feeling dismissed. However, most participants who sought care reported undergoing diagnostic evaluation. Because diagnostic evaluation was not examined according to perceived dismissal, we cannot determine whether dismissal was associated with delayed or incomplete evaluation. These findings nevertheless highlight patient-provider communication as an important component of the diagnostic experience.

Notably, fewer than half of participants were familiar with endometrial sampling. Participants insured through Medicaid were less familiar with endometrial biopsy and less likely to report that they would seek evaluation for abnormal bleeding compared with those covered by Medicare/Veterans insurance or private insurance. These observations are consistent with prior studies demonstrating disparities in access to gynecologic care by insurance status and highlight differences in knowledge and care-seeking behavior among populations already at increased risk for adverse endometrial cancer outcomes (Xu et al., 2023; Corbisiero et al., 2023).

Among participants who had not sought care, many expected bleeding to resolve spontaneously or with lifestyle changes. Structural barriers, including difficulty obtaining appointments, inability to take time away from other responsibilities, and lack of an established provider, were also reported. Learning that abnormal bleeding could represent a serious health problem and having more convenient testing options increased reported motivation to seek evaluation. Although hypothetical self-testing did not represent an existing commercial test, these findings support evaluating convenience and accessibility as patient-centered considerations in emerging diagnostic strategies.

Few studies have evaluated experiences with abnormal uterine bleeding among individuals outside the healthcare setting. By including participants who had not yet sought care, we were able to examine barriers and facilitators to diagnostic evaluation that may not be captured in clinic-based populations. Additional strengths include the use of a survey framework informed by prior semi-structured interviews with individuals who experienced AUB/PMB. These interviews guided survey development and helped identify patient-centered barriers and facilitators to evaluation. In addition, quota-based sampling was used to recruit a racially and ethnically diverse study population.

Several limitations should be considered. First, the cross-sectional design precludes causal inference, and all data were self-reported, introducing the potential for recall bias and misclassification. Although the survey was informed by qualitative interviews and piloted among interview participants, there was no formal psychometric validation. Clinical diagnoses and diagnostic evaluations were not independently verified; however, because the primary objective was to assess patient knowledge, perceptions, and experiences related to abnormal bleeding and diagnostic evaluation, participant-reported data are appropriate for assessing these outcomes. Second, participants were recruited through an opt-in online survey panel, using quota-based sampling; therefore, the sample was not nationally representative and may be subject to selection bias. Additionally, individuals without a high school diploma were excluded from participation, limiting the ability to evaluate knowledge and diagnostic experiences among populations with lower educational attainment and limiting generalizability to this group. Finally, subgroup analyses, including comparisons by PMB status, were exploratory.

## 5 Conclusion

In summary, this large and diverse study highlights important gaps in the recognition, interpretation, and evaluation of abnormal bleeding among individuals at increased risk for endometrial cancer in the United States. Delayed recognition of abnormal bleeding was common, awareness of bleeding as a potential symptom of cancer was limited, and familiarity with diagnostic evaluation was low. Misconceptions regarding the significance of AUB/PMB, reports of symptom dismissal by healthcare providers, and differences in care-seeking intentions across insurance and menopausal groups suggest that barriers to evaluation occur at multiple stages, from symptom recognition to diagnostic assessment. Despite these challenges, participants overwhelmingly identified healthcare providers as their preferred source of information, underscoring the importance of provider education, effective patient-provider communication, and public health initiatives focused on abnormal bleeding awareness. Participants also identified more convenient testing options as a potential facilitator to seeking care, suggesting that patient-centered diagnostic approaches may help reduce barriers to evaluation. By identifying where delays may occur along the pathway from symptom onset to diagnostic evaluation, this study highlights opportunities to promote earlier assessment of abnormal bleeding and improve access to timely bleeding evaluation.

**Role of the Funder:** Exact Sciences provided financial support for the study and participated in survey development. Health Advances (Boston, MA) conducted survey programming, administration, and data collection. The current analysis was conceived and initiated by the academic investigators. Statistical analyses were conducted collaboratively by the academic investigators and Exact Sciences. Academic investigators led the interpretation of the results and manuscript preparation. Exact Sciences-affiliated coauthors contributed to manuscript review. All authors approved the final manuscript and the decision to submit for publication. Exact Sciences had the opportunity to review the manuscript prior to submission but did not have authority to restrict or prevent publication. Academic investigators had access to study summaries and analytic outputs generated from the de-identified survey data but did not maintain custody of respondent-level data.

## CRediT authorship contribution statement

**Shariska P. Harrington:** Writing – review & editing, Writing – original draft, Visualization, Validation, Methodology, Investigation, Formal analysis, Conceptualization. **Negar Taheri:** Writing – review & editing, Investigation. **Manisha Salinas:** Writing – review & editing. **Andilyn Mehltretter:** Writing – review & editing, Data curation. **Nicholas McConnell:** Writing – review & editing, Data curation. **Martha O'Neill:** Writing – review & editing, Data curation. **Arushi Agarwal:** Writing – review & editing, Data curation. **Donna Hochberg:** Writing – review & editing, Data curation. **Tomasz Beer:** Writing – review & editing. **Mark Camardo:** Writing – review & editing, Visualization, Validation, Software, Formal analysis. **Elle Kielar-Grevstad:** Writing – review & editing, Resources, Project administration, Funding acquisition. **Jamie N. Bakkum-Gamez:** Writing – review & editing, Supervision, Methodology, Funding acquisition, Conceptualization.

## Ethics declaration

Written informed consent to take part in the study and to publish the article has been obtained from all participants or their legal representatives. The privacy rights of participants have been observed.

This study was performed in compliance with relevant laws, regulatory frameworks and guidelines where the research took place. This study was approved by the Advarra CIRBI Platform. (Approval No. IRB number 00078362)

## Funding

SPH is supported by the Paul Calabresi Program in Clinical/Translational Research at the Mayo Clinic Comprehensive Cancer Center [K12CA090628]. This study was funded by Exact Sciences. Exact Sciences participated in survey development and manuscript review. Health Advances (Boston, MA) (contracted by Exact Sciences), was responsible for survey administration and data collection.

## **Declaration of competing interest**

The authors declare that they have no known competing financial interests or personal relationships that could have appeared to influence the work reported in this paper.

## Appendix A Supplementary data

Supplementary material 1 Supplementary Data 1. **Full survey instrument with defined quotas.** Response formats included single-response multiple-choice questions (one answer permitted), select-all-that-apply questions, Likert-scale items, and ranking-based questions in which participants prioritized options (e.g., ranking up to three preferences). Sampling targets were established a priori to ensure representation across key demographic, clinical, and healthcare access variables. Quotas defined minimum or maximum allowable proportions within each category (e.g., age, race/ethnicity, geographic region, rural/urban classification, education, income, insurance status, menopausal status, and endometrial cancer–related characteristics). These targets were implemented during survey fielding to promote a diverse study population.

Supplementary material 2 Supplemental Data 2. **Recognition, knowledge, care-seeking, and healthcare experiences according to postmenopausal bleeding status.** Data are presented as n (%) unless otherwise specified. Participants were categorized according to whether they reported experiencing postmenopausal bleeding (PMB). Comparisons between participants with and without PMB were performed using the chi-square test or Fisher exact test, as appropriate. AUB, abnormal uterine bleeding; PMB, postmenopausal bleeding. ^1^ Recognition of AUB as a potential symptom of cancer was assessed only among participants who reported being concerned about their bleeding.

Supplementary material 3 Supplemental Data 3. **Healthcare provider type and patient-reported outcomes among participants seeking care for abnormal uterine bleeding**. Data are presented as n/N (%). Analyses were restricted to participants who reported seeking care from a healthcare provider (HCP) for abnormal uterine bleeding (AUB) (*N* = 450). For the overall distribution, percentages were calculated using all participants who sought care (*N* = 450) as the denominator. For provider concern and diagnostic evaluation outcomes, percentages were calculated within each provider type. Diagnostic evaluation represents participant-reported receipt of diagnostic evaluation for AUB. ER, emergency room; HCP, healthcare provider; AUB, abnormal uterine bleeding.
