# Supplementary material for: Barriers to the recognition and evaluation of abnormal uterine bleeding among individuals at increased risk for endometrial Cancer
Source: Gynecol Oncol Rep. 2026 Sep 24;67:102214. doi: 10.1016/j.gore.2026.102214 (PMC13636334; doi:10.1016/j.gore.2026.102214)
Supplement: Supplementary file 1 [file mmc1.pdf]

SCREENING QUESTIONS

S1 Are you currently living in the United States?

a. Yes

b. No **[THANK AND TERMINATE]**

S2 Which region of the US do you live in?

a. Northeast (Connecticut, Maine, Massachusetts, New Hampshire, New Jersey, New York, Pennsylvania, Rhode Island, Vermont)

b. South (Alabama, Arkansas, Florida, Delaware, District of Columbia, Georgia, Kentucky, Louisiana, Maryland, Mississippi, North Carolina, Oklahoma, South Carolina, Tennessee, Texas, Virginia, West Virginia)

c. Midwest (Illinois, Indiana, Iowa, Kansas, Michigan, Minnesota, Missouri, Nebraska, North Dakota, Ohio, South Dakota, Wisconsin)

d. West (Alaska, Arizona, California, Colorado, Hawaii, Idaho, Montana, Nevada, New Mexico, Oregon, Utah, Wyoming, excluding Washington)

e. Washington state **[THANK AND TERMINATE]**

[QUOTA: <35% OF RESPONDENTS FROM NORTHEAST, WEST, SOUTH, MIDWEST]

S3 How would you describe the area where you live?

a. Urban (living within 5 miles from a major city center)
b. Suburban (living more than 5 miles and less than 25 miles from a major city center)
c. Rural (living more than 25 miles from a major city center)

[QUOTA: >20% FROM RURAL, <50% OF RESPONDENTS FROM EACH AREA]

S4 How old are you?

a. 0-18 years old **[THANK AND TERMINATE]**
b. 19-29 years old **[THANK AND TERMINATE]**
c. 30-39 years old
d. 40-44 years old
e. 45-51 years old
f. 52-59 years old
g. 60-70 years old
h. 71-79 years old
i. 80 years or older **[THANK AND TERMINATE]**

[QUOTA: <25% OF AGE 30-39, <25% OF AGE 40-44, <45% OF AGE 45-50, <45% of AGE 50-70, <5% 71-79]

S5 Which racial or ethnic group do you most closely identify with?

a. American Indian or Alaskan Native
b. Asian
c. Black or African American
d. Hispanic or Latino
e. Middle East or North Africa
f. Native Hawaiian or Other Pacific Islander
g. White/Caucasian
h. Multiple Ethnicity/Other
i. Prefer not to disclose **[TERMINATE AND THANK]**

[QUOTA: 20% AFRICAN AMERICAN, 20% ASIAN, 20% HISPANIC, 20% WHITE, AND 20% ALL OTHER RESPONDENT GROUPS]

S6 What religion do you most closely identify with?

a. Buddhism
b. Confucianism
c. Islam
d. Judaism
e. Catholicism
f. Christianity
g. Hinduism
h. Mormonism
i. Agnosticism
j. Atheism
k. Other (please specify): **[FORCE TEXT]**
l. Prefer not to disclose

[QUOTA: >10% ISLAM]

S7 What is the highest level of education you have completed?

a. High school diploma or GED

b. Associate's degree

c. Bachelor's degree

d. Graduate degree

e. None of the above **[THANK AND TERMINATE]**

[QUOTA: <50% OF ALL RESPONDENT GROUPS]

S8 How would you describe your current employment status?

a. Employed full-time (35 hours a week or more)
b. Employed part-time (less than 35 hours a week)
c. Self-employed
d. Unemployed and looking for work
e. Unemployed and not currently looking for work
f. Unpaid caregiver (e.g., stay-at-home mom)
g. Student
h. Retired
i. Unable to work (e.g., disabled)

[QUOTA: <50% OF ALL RESPONDENT GROUPS]

S9 What is your annual household income?

Please include the combined income (before taxes) from all members of your household.

a. Less than $25,000
b. $25,001-75,000
c. $75,001-125,000
d. $125,001-175,000
e. $175,001-225,000
f. $225,001-275,000
g. $275,001-325,000
h. Greater than $325,000

[QUOTA: <50% OF ALL RESPONDENT GROUPS]

S10 What type of health insurance do you have?

a. Private insurance (e.g., Blue Cross, UnitedHealthCare)
b. Medicare and/or Medicare Advantage
c. Medicare and Private insurance
d. Medicare and Medicaid
e. Medicaid only
f. Veteran's Administration or Other Government Insurance
g. Uninsured/Self-insured

[QUOTA: <40% OF RESPONDENTS OF PRIVATE (A), PUBLIC (B-F), AND UNINSURED (G)]

S11 Do you still have a period?

Medication, like the birth control pill, can temporarily stop your period. When answering about whether or not you still get your period, please consider, if you were off the medication would you still be getting your period?

a. Yes, I get my period once a month

b. Yes, I get my period more than once a month

c. Yes, I get my period every 2-6 months

d. Yes, I get my period every 6-12 months

e. No, I have not had my period for more than 12 months

f. Not applicable – I have never had a period due to my sex **[THANK AND TERMINATE]**

S12 **[IF “NO PERIOD FOR 12 MONTHS” IS SELECTED]** Why have you not had your period in more than 12 months?

Medication, like the birth control pill, can temporarily stop your period. When answering about why you have not had your period, please consider, if you were off the medication would you still be getting your period?

a. I had uterine surgery (e.g., hysterectomy or endometrial ablation) and no longer have a period

b. I have gone through menopause

c. I am currently going through menopause. I have stopped having monthly periods and am experiencing symptoms like hot flashes and mood swings

d. I don’t know why

e. Other (please specify): **[FORCE TEXT]**

[QUOTA: <20% PREMENOPAUSE (PERIOD WITHIN LAST 12 MONTHS), <50% OF MENOPAUSE (CURRENTLY GOING THROUGH, DON’T KNOW, OTHER), AND <60% OF POST-MENOPAUSE (UTERINE SURGERY, GONE THROUGH)]

S13 Which of the following gynecological symptoms have you experienced? Your experience can be current or in the past. Please select all that apply.

Gynecological symptoms are anything related to your female reproductive organs, i.e., your vagina, cervix, uterus, ovaries, and fallopian tubes.

a. Vaginal bleeding in between periods (include any bleeding between periods, such as spotting, light pink or brown blood, and bleeding after intercourse)

b. Heavy vaginal bleeding (needing to change a tampon/pad every 1-2 hours or bleeding lasting longer than 8 days)

c. Vaginal bleeding after menopause (include any bleeding after menopause such as spotting, light pink or brown blood, and bleeding after intercourse)

d. Red, brown, or pink colored discharge

e. None of the above **[TERMINATE AND THANK]**

S14 Have you received a diagnosis for any of the following gynecological conditions? Your diagnosis can either be current or in the past. Please select all that apply.

a. Diagnosed abnormal uterine bleeding

b. Uterine polyps or fibroids

c. Endometrial hyperplasia (a condition in which there is an irregular thickening of lining of the uterus and is not equal to endometriosis)

d. Endometrial cancer (a type of cancer that begins as a growth of cells in the uterus)

e. None of the above **[EXCLUSIVE OPTION]**

[SELECT ALL THAT APPLY]

[QUOTA: >10% ENDOMETRIAL CANCER, <90% ALL OTHER GROUPS]

S15 Have you ever talked to a healthcare provider (e.g., doctor or nurse) for the gynecological symptoms or conditions you have experienced?

a. Yes

b. No

[QUOTA: <70% SEEN HCP FOR BLEEDING (YES SEEN HCP OR DIAGNOSIS OF CONDITION), <40% NOT SEEN HCP FOR BLEEDING]

S16 **[IF "SEEN HCP" IS SELECTED]** Have you received test(s) to determine the cause of the gynecological symptoms or conditions you have experienced?

a. Yes

b. No

[QUOTA: <20% HAVE NOT RECEIVED TEST (NO TEST AND NO DIAGNOSIS OF CONDITION)]

S17 **[IF “RECEIVED TESTING” IS SELECTED]** When did you receive test(s) to determine the cause of your abnormal vaginal bleeding or bleeding that happened after menopause?

a. Less than 1 year ago

b. 1-2 years ago

c. 3-4 years ago

d. 5-6 years ago

e. More than 7 years ago **[THANK AND TERMINATE UNLESS ENDOMETRIAL CANCER AND HYPERPLASIA]**

S18 Have you used or are you currently using estrogen therapy?

Estrogen therapy is a treatment used to help severe menopausal symptoms. It does not include treatments like the birth control pill where estrogen is used in combination with progestin.

a. Yes

b. No

[INFORM RISK FACTOR QUOTA: <70% HAVE AT LEAST 1 RISK, <40% HAVE NO RISKS]

[INFORM TERMINATE AND THANK: IF <45 YEARS OLD AND HAVE NO RISKS]

S19 Have you used or are you currently using tamoxifen?

Tamoxifen is a treatment for breast or ovarian cancer.

a. Yes

b. No

[INFORM RISK FACTOR QUOTA: <70% HAVE AT LEAST 1 RISK, <40% HAVE NO RISKS]

[INFORM TERMINATE AND THANK: IF <45 YEARS OLD AND HAVE NO RISKS]

S20 Have you ever been diagnosed with polycystic ovary syndrome (PCOS)?

a. Yes

b. No

[INFORM RISK FACTOR QUOTA: <70% HAVE AT LEAST 1 RISK, <40% HAVE NO RISKS]

[INFORM TERMINATE AND THANK: IF <45 YEARS OLD AND HAVE NO RISKS]

S21 Do you currently have obesity or high body fat, or history of obesity or high body fat?

One way to measure obesity or high body fat is the Body Mass Index (BMI) score. If you are aware of your BMI score, please consider obesity or high body fat as a Body Mass Index (BMI) of more than 30.

a. Yes

b. No

[INFORM RISK FACTOR QUOTA: <70% HAVE AT LEAST 1 RISK, <40% HAVE NO RISKS]

[INFORM TERMINATE AND THANK: IF <45 YEARS OLD AND HAVE NO RISKS]

S22 Do you have a family history of any of the following cancers? Please select all that apply.

Family history means someone in your family, who you have a blood relationship with, has had the condition. Do not consider family members who are related to you by marriage or adoption, for example your spouse.

a. Breast cancer

b. Ovarian cancer

c. Endometrial cancer

d. Colon cancer

e. I don't know

f. No, I do not have a family history of these cancers

[INFORM RISK FACTOR QUOTA: <70% HAVE AT LEAST 1 RISK, <40% HAVE NO RISKS]

[INFORM TERMINATE AND THANK: IF <45 YEARS OLD AND HAVE NO RISKS]

Congratulations! You are invited to participate in a web-based online survey on diagnostic testing for abnormal uterine bleeding/postmenopausal bleeding and endometrial cancer. This survey will take approximately 20 minutes to complete.

We are conducting this survey to support the development of a publication to generate awareness of abnormal uterine bleeding/post-menopausal bleeding and endometrial cancer testing.

Your participation in this survey is strictly voluntary. You are free to withdraw your consent and to discontinue participation in this survey at any time without prejudice. You may refuse to take part in the research or exit the survey at any time without penalty. However, compensation will not be given to participants who withdraw from this survey.

You will be paid an honorarium by the sampling provider, Dynata, for completion of the survey by May 3rd, 2024. Your responses may help us learn more about varying perspectives on seeking care for abnormal uterine bleeding/postmenopausal bleeding, current testing options for the relevant conditions, and responses to an alternative test in development for endometrial cancer. The possible risks of the study are minimal. You may feel a little discomfort answering personal survey questions about your experiences and/or opinions.

All information collected will be used only for this research and will be kept confidential. There will be no connection to you specifically in the results or in future publication of the results. All answers are reported in an aggregate manner.

By clicking START SURVEY you are verifying that you are 21 years or older, have read the explanation of the study, that you agree to the collection of your personal information, and agree to participate in this study. You also understand that your participation in this study is strictly voluntary.

Thank you for your participation.

ABNORMAL BLEEDING CARE SEEKING BEHAVIORS

1. Do you have a family history of any of the following conditions? Please select all that apply.

Family history means someone in your family, <u>who you have a blood relationship with</u>, has had the condition. Do <u>not</u> consider family members who are related to you by marriage or adoption, for example your spouse.

   a. Irregular vaginal bleeding in between periods
   b. Abnormally heavy vaginal bleeding during or in between periods
   c. Vaginal bleeding after menopause
   d. Uterine polyps or fibroids
   e. Endometrial hyperplasia
   f. No **[EXCLUISVE OPTION]**
   g. I don’t know **[EXCLUISVE OPTION]**

[SELECT ALL THAT APPLY]

2. Do you have any chronic diseases or illnesses that you see a doctor regularly for? Please select all that apply.

   Please consider a chronic disease to be a condition that lasts 1 year or more and requires ongoing medical care.

   a. Amyotrophic Lateral Sclerosis (ALS) or Lou Gehrig's Disease
   b. Alzheimer's Disease or other dementias
   c. Arthritis
   d. Asthma
   e. Cancer other than breast or ovarian
   f. Chronic Obstructive Pulmonary Disease (COPD)
   g. Crohn's Disease, Ulcerative Colitis, Other Inflammatory Bowel Diseases, or Irritable Bowel Syndrome
   h. Cystic Fibrosis
   i. Diabetes
   j. Heart Disease
   k. Osteoporosis
   l. Other (please specify): **[FORCE RESPONSE]**
   m. No

[SELECT ALL THAT APPLY]

3. Have you ever given birth or carried a pregnancy to full term?
    a. Yes
    b. No

4. What type of doctor or healthcare provider do you typically see for your routine gynecological care?

Gynecological is anything related to your female reproductive organs, i.e., your vagina, cervix, uterus, ovaries, and fallopian tubes. Routine care may include pelvic exams, pap tests, cancer screenings, treatment of vaginal infections, and more.

   a. Primary care provider (primary care physician, physician assistant, or nurse practitioner)
   b. Gynecologist / Obstetrician Gynecologist (Ob-Gyn)
   c. Other (please specify): **[FORCE TEXT]**
   d. I do not receive routine gynecological care

5. **[IF “I DO NOT RECIVE CARE” IS NOT SELECTED]** To which gender identity does your doctor or healthcare provider you see for your routine gynecological care identify with?

    a. Female

    b. Male

    c. Nonbinary

6. What gender of doctor would you prefer to see for your routine gynecological care?
    a. Female
    b. Male
    c. Nonbinary
    d. No preference

7. **[IF "PCP OR GYN" IS SELECTED]** How often do you see this doctor?

    a. Multiple times per year

    b. Once a year

    c. Less than once a year

9. **[IF “PCP OR GYN” IS SELECTED]** What are these doctors’ visits for? Please select all that apply.

   a. Annual check-up

   b. Prescriptions

   c. Condition you have had for a year or longer (i.e., chronic illness)

   d. Symptoms or illness you have had for a short time (i.e., acute illness)

   e. Tests to check for common diseases even when you don’t have symptoms (i.e., screening tests like pap smear or breast exam)

   f. Other (please specify): **[FORCE TEXT]**

[SELECT ALL THAT APPLY]

10. What feminine hygiene products do/did you prefer to use when you have/had your period?

   a. Tampon
   b. Diva cup
   c. Pad
   d. Period underwear
   e. Other (please specify): **[FORCE TEXT]**

11. How often do/did you use tampons?

If you no longer get a period, please consider your use when you had a period.

a. Frequently use - it's the primary feminine hygiene product I use for my period
b. Sometimes use - I use when my primary feminine hygiene product is not available
c. Rarely use - I use only when needed and I have no other options
d. Never use - I have never used a tampon before

12. **[IF "TAMPON PREFER PRODUCT" IS NOT SELECTED]** Why are/were tampons not your preferred and/or primary feminine hygiene product for your period? Please select all that apply.

If you no longer get a period, please consider your use when you had a period.

a. Bad personal experience
b. Friends/family had bad experience
c. My family/friends don't use them
d. They leak/are not absorbent enough
e. Painful
f. Uncomfortable
g. Fear
h. Risk of toxic shock syndrome (TSS)
i. Gross
j. Cost
k. Cultural/religious reasons
l. Other (please specify): **[FORCE TEXT]**

13. You said you are currently experiencing or have a history of **[PIPE BLEEDING SYMPTOMS]**, which is considered **abnormal vaginal bleeding**. The remainder of this survey will be focused on your experience with abnormal vaginal bleeding.

14. How much time passed before you realized the vaginal bleeding you were experiencing was not normal?

a. I immediately realized it was abnormal
b. Less than 1 month
c. 1 to 6 months
d. 7 to 11 months
e. 1 to 2 years
f. 2 to 3 years
g. More than 3 years

15. What did you think the abnormal vaginal bleeding could mean? Please select all that apply.

    a. There is something wrong

    b. This is not something to worry about

[SELECT ALL THAT APPLY]

16. **[IF “SOMETHING IS WRONG” IS SELECTED]** What did you think was wrong about the abnormal vaginal bleeding? Please select all that apply.

  a. There’s a problem with my period

  b. It happens to people my age and may be a problem

  c. It is a symptom of a reproductive organ (i.e., vagina, uterus) related condition

  d. It is a symptom of cancer

  e. It runs in my family and was a problem

  f. Other (please specify): **[FORCE TEXT]**

17. **[IF “SYMPTOM OF CANCER” IS SELECTED]** What cancer did you think it was a symptom for? Please select all that apply.

   a. Ovarian cancer

   b. Cervical cancer

   c. Vaginal cancer

   d. Endometrial cancer

   e. Bladder cancer

   f. Other (please specify): **[FORCE TEXT]**

   g. I did not know which type of cancer **[EXCLUSIVE OPTION]**

18. **[IF "SYMPTOM OF EC" IS SELECTED]** How did you learn that abnormal vaginal bleeding is a symptom of endometrial cancer? Please select all that apply.

   a. Conversation with my doctor
   b. Message from my insurance company (e.g., email, brochure, or mail)
   c. Message from my pharmacy (e.g., email, brochure, or mail)
   d. Conversations with friends and family
   e. Online research
   f. Radio or TV
   g. Social Media
   h. Community event (e.g., event at church, walk for breast cancer awareness)
   i. Other (please specify): **[FORCE TEXT]**

[SELECT ALL THAT APPLY]

19. **[IF "NOTHING TO WORRY ABOUT" IS SELECTED]** Why did you think the abnormal vaginal bleeding was not something to worry about? Please select all that apply.

   a. It will go away on its own
   b. It has been like this since I first got my period
   c. It's normal in my family
   d. It's normal for people my age
   e. It is caused by stress
   f. It is caused by hormones
   g. It is caused by my weight
   h. It is only inconvenient and does not need to be addressed
   i. Other (please specify): **[FORCE TEXT]**

20. What impact did your abnormal vaginal bleeding have on your quality of life? Please rate the following with 1 being not impacted at all and 5 being extremely impacted.

	Not at all impacted (1)	Somewhat impacted (2)	Slightly impacted (3)	Very impacted (4)	Extremely impacted (5)
My ability to go to work	[RADIO BUTTONS]				
My ability to complete errands (e.g., grocery shopping)					
My ability to enjoy social gatherings					
My ability to exercise					
My ability to support my friends and family					
My ability to engage in intimate relationships					
My mental health					

21. **[IF “I DO NOT RECIVE CARE” IS NOT SELECTED]** Did your doctor ever ask you about the frequency or heaviness of your period without you bringing it up yourself?

   a. Yes

   b. No

22. What have you done to address your abnormal vaginal bleeding? Please select all that apply.

   a. Online research (e.g., Google)
   b. Social media search (e.g., Facebook)
   c. Discussed with my family / friends
   d. Discussed with my doctor
   e. Discussed with a provider other than my doctor
   f. Other (please specify): **[FORCE TEXT]**
   g. I did not do anything **[EXCLUSIVE OPTION]**

[SELECT ALL THAT APPLY]

23. **[IF "DISCUSSED WITH FAMILY / FRIENDS" IS SELECTED]** What did your friends / family tell you about the abnormal vaginal bleeding? Please select all that apply.

    a. The abnormal bleeding is concerning
    b. The abnormal bleeding is not concerning
    c. I should go see my doctor
    d. The abnormal bleeding would go away on its own
    e. The abnormal bleeding is normal at this age
    f. The abnormal bleeding is normal and runs in the family
    g. The abnormal bleeding is normal and caused by stress
    h. Other (please specify): **[FORCE TEXT]**
    i. They did not know what I should do **[EXCLUSIVE OPTION]**

[SELECT ALL THAT APPLY]

24. Are you part of any online support community focused on abnormal vaginal bleeding (e.g., Facebook groups, Reddit pages)?

    a. Yes

    b. No

25. **[IF “SEEN HCP FOR ABNORMAL BLEEDING” IS SELECTED]** Where did you go to discuss with a doctor about your abnormal vaginal bleeding?

   a. My regular primary care provider office or online (e.g., via Zoom)
   b. My regular gynecologist / Ob-Gyn office or online (e.g., via Zoom)
   c. A new primary care provider office or online (e.g., via Zoom)
   d. A new gynecologist / Ob-Gyn office or online (e.g., via Zoom)
   e. Urgent care or walk-in clinic
   f. Community or public health clinic
   g. Retail clinic (e.g., CVS minute clinic)
   h. Emergency room or hospital
   i. Telehealth (i.e., met with a doctor online who is not my primary care provider or gynecologist)
   j. Other (please specify): **[FORCE TEXT]**

26. **[IF "DISCUSSED AT PRIMARY CARE OR GYN OFFICE" IS SELECTED]** At what type of appointment did you discuss the abnormal vaginal bleeding with your doctor?

    a. My annual check-up

    b. An appointment that I already had scheduled for a different reason

    c. An appointment I scheduled specifically to discuss abnormal vaginal bleeding

27. **[IF "SEEN HCP FOR ABNORMAL BLEEDING" SELECTED]** How long did you wait to discuss with your doctor after you realized the vaginal bleeding was abnormal?

    a. Less than 1 week
    b. 1 week to 1 month
    c. 1 to 2 months
    d. 3 to 6 months
    e. 7 months to 1 year
    f. More than 1 year

28. **[IF “SAW DOCTOR AFTER 3 MONTHS TO MORE THAN 1 YEAR IS SELECTED]** Why didn’t you go see a doctor right away? Please select all that apply.

   a. I could not take time away from work, family, or other responsibilities

   b. **[IF “DISCUSSED AT APPOINTMENT ALREADY SCHEDULE OR ANNUAL CHECK UP” IS SELECTED]** I already had an existing appointment scheduled for later in the year so I waited until then

   c. It was hard to book an appointment with a doctor

   d. I was not able to travel to an appointment

   e. **[IF “PCP OR GYN” IS NOT SELECTED]** I do not have a PCP or gynecologist

   f. I did not think it was a problem

   g. I was worried about the cost

   h. I was worried about insurance coverage

   i. I was worried about what the doctor would say or do

   j. I had a previous negative experience with the healthcare system

   k. Other (please specify): **[FORCE TEXT]**

[SELECT ALL THAT APPLY]

29. [IF "SEEN HCP FOR ABNORMAL BLEEDING" IS SELECTED] Why did you decide to go see your doctor for your abnormal vaginal bleeding?

Please rank **up to 3** of the following reasons. Drag and drop reasons from left to right, with the most influential reason first.

	**Rank Reasons You Went to Doctor**
The bleeding was painful	
The bleeding was heavy	
The bleeding was impacting my daily routine	
The bleeding was inconvenient	
I was worried there was something wrong	
I was worried I had cancer	
My friends / family told me to	
My doctor told me to see them if I ever experienced abnormal vaginal bleeding	
The internet told me to see my doctor if I ever experienced abnormal vaginal bleeding	
Other (please specify) [FORCE TEXT]	

30. **[IF "SEEN HCP FOR ABNORMAL BLEEDING" IS SELECTED]** What did your doctor tell you the abnormal bleeding could mean? Please select all that apply.

    a. It is a sign of endometrial cancer
    b. It is a sign of cervical cancer
    c. It is a sign of pre-cancer (e.g., uterine, cervical or vaginal pre-cancer)
    d. It is a sign of infection
    e. It is a sign of hormonal imbalance (e.g., PCOS)
    f. It is a sign of non-cancerous growths (e.g., polyps, fibroids)
    g. It is caused by medications and/or vaccines (e.g., hormonal birth control pill)
    h. It is caused by stress
    i. It is caused by weight
    j. It is caused by pregnancy
    k. It is normal for your age
    l. It is not a problem
    m. Other (please specify): **[FORCE TEXT]**
    n. They did not say what it could mean **[EXCLUSIVE OPTION]**

[SELECT ALL THAT APPLY]

31. **[IF “SIGN OF CANCER, INFECTION, PCOS, POLYPS” IS NOT SELECTED]** What did you do when your doctor was not concerned about your abnormal vaginal bleeding? Please select all that apply.

   a. Told them I am concerned and would like to fix it

   b. Discussed with friends and family

   c. Discussed with a different doctor

   d. Researched the symptoms on my own

   e. Trusted it was okay and did not worry about it **[EXCLUSIVE OPTION]**

   f. Other (please specify): **[FORCE TEXT]**

[SELECT ALL THAT APPLY]

32. **[IF “SEEN HCP FOR ABNORMAL BLEEDING” IS SELECTED]** How many times have you discussed your abnormal vaginal bleeding with a doctor?

   a. Once
   b. 2 to 4 times
   c. 5 to 7 times
   d. 8 to 10 times
   e. More than 10 times

33. **[IF "NOT SEEN HCP FOR ABNORMAL BLEEDING" IS SELECTED]** How long has it been since you first realized your vaginal bleeding was abnormal?

   a. Less than 1 month

   b. 1 to 6 months

   c. 7 to 11 months

   d. 1 to 2 years

   e. More than 2 years

34. [IF "NOT SEEN HCP FOR ABNORMAL BLEEDING" IS SELECTED] Why haven't you seen a doctor for your abnormal vaginal bleeding?

Please rank **up to 3** of the following reasons. Drag each reason from left to right, starting with the most important reason.

	**Rank Reasons for Not Seeing Doctor**
I cannot/have not yet been able to get an appointment with my doctor	
[IF "PCP OR GYN" IS NOT SELECTED] I do not have a primary care provider or gynecologist	
I cannot take time away from work, family or other responsibilities	
I cannot travel to an appointment	
I don't trust healthcare providers	
I am afraid of healthcare providers	
I am uncomfortable receiving care from my doctor because of their gender	
I am afraid of getting a pelvic exam	
I am afraid of getting other tests	
I am afraid of getting treatment (i.e., medication or surgery)	
I am afraid of getting bad results that tell me I have a disease	
I don't want to pay for it	

I am worried my insurance won’t cover it	
Abnormal bleeding is not something to worry about	
I was waiting to see if it would go away on its own	
I was waiting to see if it would go away with lifestyle changes	
Other (please specify): **[FORCE TEXT]**	

35. **[IF “<u>NOT</u> SEEN HCP FOR ABNORMAL BLEEDING” IS SELECTED]** How likely are you to see a doctor in the future for abnormal vaginal bleeding?

a. Extremely likely

b. Very likely

c. Somewhat likely

d. Slightly likely

e. Not at all likely

36. **[IF "EXTREMELY LIKELY TO SLIGHTLY LIKELY SEE DOCTOR" IS SELECTED]** Where will you go to see the doctor for your abnormal vaginal bleeding?

a. My primary care providers office or online visit (e.g., Zoom)

b. My gynecologist / Ob-Gyn office or online visit (e.g., Zoom)

c. Urgent care or walk-in clinic

d. Retail clinic (e.g., CVS minute clinic)

e. Emergency room or hospital

f. An office or telehealth visit with a doctor that is not my regular provider

g. Other (please specify): **[FORCE TEXT]**

37. **[IF “NOT AT ALL LIKELY SEE DOCTOR” IS SELECTED]** Are you still experiencing abnormal vaginal bleeding?

a. Yes, I often have abnormal vaginal bleeding (i.e., more than 12 times per year)

b. Yes, I occasionally have abnormal vaginal bleeding (i.e., 6 to 12 times per year)

c. Yes, I sometimes have abnormal vaginal bleeding (i.e., 1 to 5 times per year)

d. Yes, I rarely have abnormal vaginal bleeding (i.e., less than once per year)

e. No, I have not had abnormal vaginal bleeding since

38. **[IF "NOT SEEN HCP FOR ABNORMAL BLEEDING" IS SELECTED]** What would motivate you to see a doctor for your abnormal vaginal bleeding? Please rate the following factors, with 1 being would not motivate me at all and 5 being extremely motivating.

	Would not motivate me at all (1)	Slightly motivating (2)	Somewhat motivating (3)	Very motivating (4)	Extremely motivating (5)
There is more blood when I have abnormal vaginal bleeding	**[RADIO BUTTONS]**				
Abnormal vaginal bleeding happens more frequently					
I experience other symptoms that I think are related to my reproductive organs (e.g., cramps)					
I learn that abnormal bleeding could be a serious problem					
I am able to get an appointment with my doctor					
The doctor I'm seeing is my preferred gender of doctor					
There are new affordable testing options					
There are new more convenient testing options					

There are tests I can do by myself (i.e., self-testing like COVID-19)	

39. How would you most prefer to learn about the causes of abnormal vaginal bleeding?

Please rank **up to 3** options. Drag each reason from left to right, starting with the method that would be the most helpful.

	Rank Methods For Learning
Conversations with my doctor	
Brochures from my doctor	
Brochures from my pharmacy	
Brochures from my insurance	
TV or radio commercials	
Social media ads	

40. The following are descriptions of tests that can determine the cause of abnormal vaginal bleeding. Please take a moment to review the different test methods.

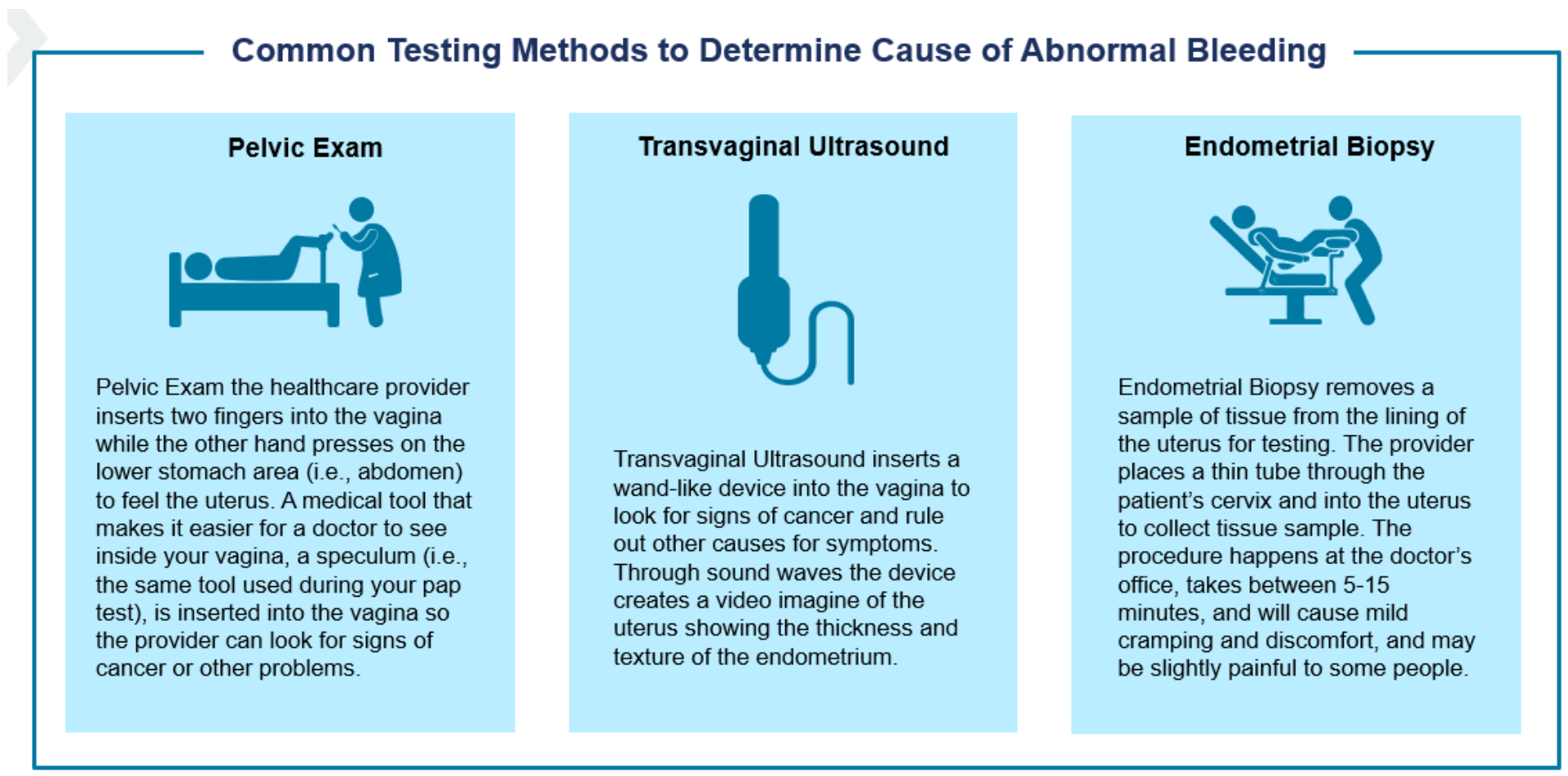

Source: Health Advances analysis, Dana-Farber, Cleveland Clinic, Mayo Clinic.

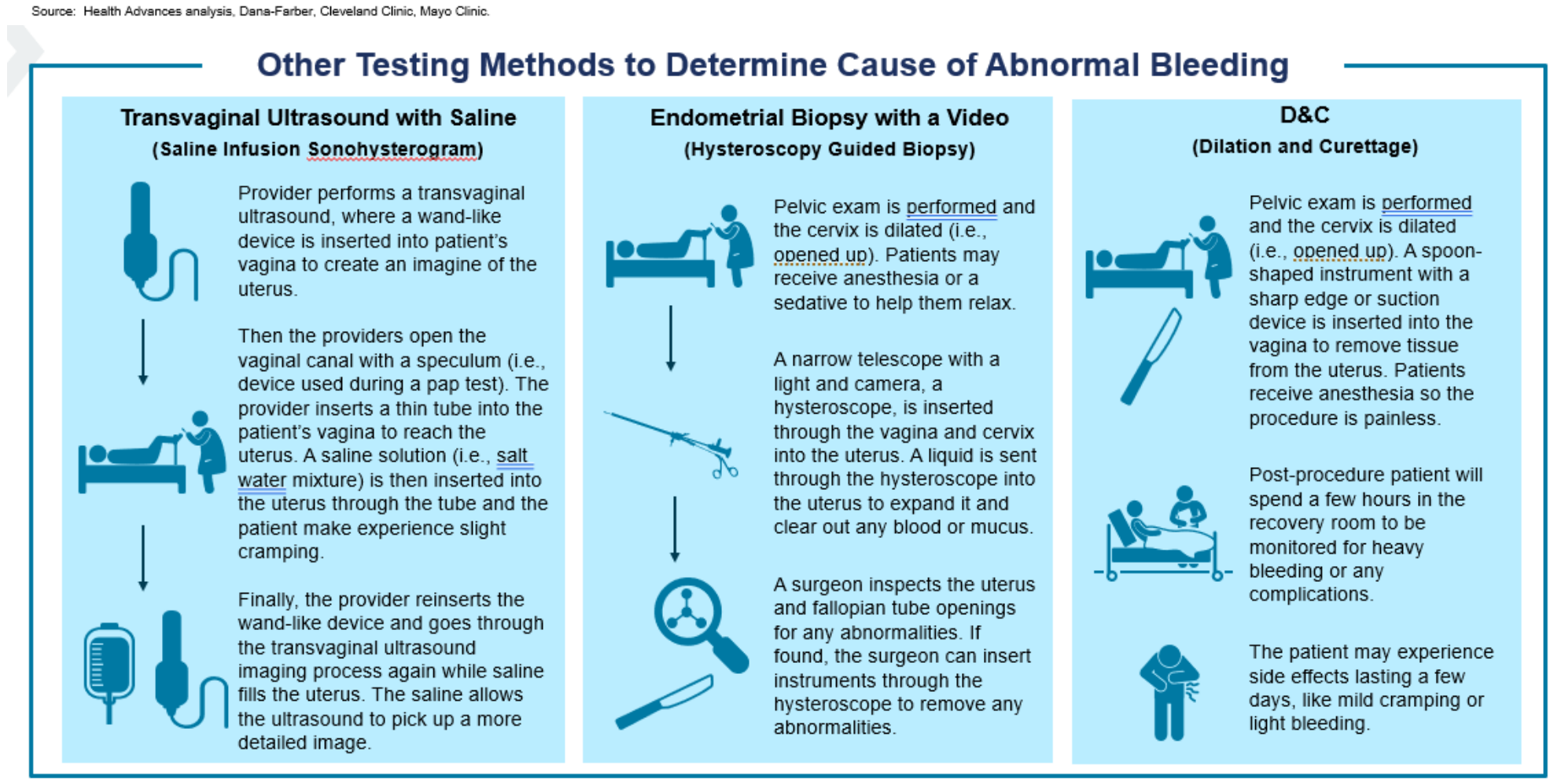

Source: Health Advances analysis, Dana-Farber, Cleveland Clinic, Mayo Clinic.

Other Testing Methods to Determine Cause of Abnormal Bleeding

Pregnancy Blood Test
(Human chorionic gonadotropin blood test)

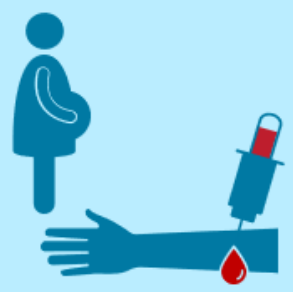

Blood test that measures the specific level of HCG in the blood. HCG is a hormone produced in the body during pregnancy.

Hormone Panel Blood Test

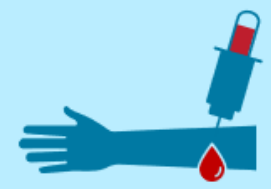

Blood test that measures the specific level of estrogen, progesterone, follicle-stimulating hormone (FSH), testosterone, thyroid hormones, and luteinizing hormone. These tests can detect fertility problems, where you are in your menstrual cycle, or whether you’re starting menopause.

41. **[IF “SEEN HCP FOR BLEEDING” IS SELECTED]** Which tests for abnormal vaginal bleeding did you know of before you discussed your abnormal vaginal bleeding with your doctor? Please select all that apply.

[LINK TO DESCRIPTIONS]

a. Pelvic exam

b. Transvaginal ultrasound

c. Saline infusion sonohysterogram (i.e., transvaginal ultrasound with saline)

d. Endometrial biopsy

e. Hysteroscopy guided biopsy (i.e., endometrial biopsy with a camera)

f. D&C (Dilation and curettage)

g. Pregnancy blood test

h. Hormone panel blood test

i. I don’t know

j. None of the tests

[SELECT ALL THAT APPLY]

42. **[IF "TEST AWARENESS PRE-DOCTOR DISCUSSION" IS SELECTED]** How did you learn about these tests for abnormal vaginal bleeding before you discussed your abnormal vaginal bleeding with your doctor? Please select all that apply.

   a. Online research
   b. Discussion with friends / family
   c. TV or radio
   d. Social media ads
   e. Conversations with my healthcare provider before I experienced abnormal vaginal bleeding
   f. Brochure at doctor's office
   g. Brochure from pharmacy
   h. Brochure from insurance
   i. Other (please specify): **[FORCE TEXT]**

43. **[IF “SEEN HCP FOR ABNORMAL BLEEDING” IS SELECTED]** How many times did you discuss your abnormal vaginal bleeding symptoms with your doctor until they discussed testing with you?

   a. After the first discussion
   b. After 2 to 3 discussions
   c. After 4 to 6 discussions
   d. After 7 to 9 discussions
   e. After 10 or more discussions
   f. My doctor has not discussed testing

44. **[IF “DOCTOR DISCUSSED TESTING” IS SELECTED]** What tests for your abnormal vaginal bleeding has your doctor discussed with you? Please select all that apply.

[LINK TO DESCRIPTIONS]

a. Pelvic exam

b. Transvaginal ultrasound

c. Saline infusion sonohysterogram (i.e., transvaginal ultrasound with saline)

d. Endometrial biopsy

e. Hysteroscopy guided biopsy (i.e., endometrial biopsy with a camera)

f. D&C (Dilation and curettage)

g. Pregnancy blood test

h. Hormone panel blood test

i. I don’t know

j. None of the described tests

[SELECT ALL THAT APPLY]

45. **[IF "SEEN HCP FOR ABNORMAL BLEEDING" IS SELECTED]** Did you complete all test(s) your doctor discussed with you for your abnormal vaginal bleeding?

   c. Yes, I completed all tests they discussed

   d. No, I only completed some of the tests they discussed

   e. No, I did not receive any tests

46. **[IF "COMPLETED SOME TESTS" IS SELECTED]** Which test(s) for your abnormal vaginal bleeding did you complete? Please select all that apply.

[LINK TO DESCRIPTIONS]

[DISPLAY TEST IF DISCUSSED BY DOCTOR OR I DON'T KNOW]

a. Pelvic exam
b. Transvaginal ultrasound
c. Saline infusion sonohysterogram (i.e., transvaginal ultrasound with saline)
d. Endometrial biopsy
e. Hysteroscopy guided biopsy (i.e., endometrial biopsy with a camera)
f. D&C (Dilation and curettage)
g. Pregnancy blood test
h. Hormone panel blood test
i. I don't know

[SELECT ALL THAT APPLY]

47. **[IF “I DON’T KNOW” IS SELECTED]** How many tests have you received for your abnormal vaginal bleeding?

a. 1
b. 2
c. 3
d. 4
e. 5
f. 6
g. More than 6
h. I don’t remember

48. **[IF "I DON'T KNOW" IS SELECTED]** Were you sedated / put to sleep for any of the test(s) for your abnormal vaginal bleeding?

   a. Yes

   b. No

49. **[IF "RECEIVED TEST" IS SELECTED]** Did the first doctor you discussed your abnormal vaginal bleeding with perform the test(s) you completed?

   a. Yes

   b. No

50. **[IF “RECEIVED TEST” IS SELECTED]** How many doctors did you see to complete testing to determine the cause of your abnormal vaginal bleeding?

a. 1

b. 2

c. 3

d. 4

e. 5

f. 6

g. 7

h. 8 or more

51. **[IF “RECEIVED TEST” IS SELECTED]** How many separate appointments did you have to complete testing to determine the cause of your abnormal vaginal bleeding?

a. 1
b. 2
c. 3
d. 4
e. 5
f. 6
g. 7
h. 8 or more

52. **[IF “RECEIVED TEST” IS SELECTED]** How involved were you in the decision of which test you received for determining the cause of your abnormal bleeding?

a. My doctor gave me complete control over which test(s) I received

b. My doctor presented the test(s) and I made the final decision of which I received

c. My doctor presented the test(s) and discussed my preferences, but my doctor made the final decision

d. My doctor told me the tests I would receive, I was not involved in the decision

53. **[IF “RECEIVED TEST” IS SELECTED]** How satisfied were you with the testing process?

If you are still undergoing testing please consider your level of satisfaction to date.

a. Extremely satisfied
b. Very satisfied
c. Somewhat satisfied
d. Slightly satisfied
e. Not at all satisfied

54. [IF “RECEIVED TEST” IS SELECTED] What have you disliked most about the test(s) you received for your abnormal vaginal bleeding?

Please rank **up to 3** options. Drag each reason from left to right, starting with the thing you disliked the most.

[LINK TO DESCRIPTIONS]

	**Most Disliked**
Invasiveness of test(s)	
Discomfort I experienced during test	
Pain / side effects I experienced after test	
Amount of time the test took	
Amount of time it took me to receive the results of the test	[RANK TOP 3]
Results were not able to determine the cause of the abnormal bleeding (i.e., the test was inconclusive)	
Amount covered by insurance	
Cost of test(s)	
Challenges in scheduling of test(s)	
Lack of availability of doctors that can perform test(s)	
I did not dislike anything about the test [EXCLUSIVE]	

55. [IF "TEST RECEIVED" IS SELECTED] How would you rate the <u>discomfort you experienced during</u> the following tests? Please rate the discomfort of the following tests, with 1 being no discomfort and 5 high discomfort.

[LINK TO DESCRIPTIONS]

[DISPLAY ONLY TESTS RECEIVED]	No discomfort (1)	(2)	Moderate discomfort (3)	(4)	High discomfort (5)
Pelvic exam	[RADIO BUTTONS]				
Transvaginal ultrasound					
Saline infusion sonohysterogram (i.e., transvaginal ultrasound with saline)					
Endometrial biopsy					
Hysteroscopy guided biopsy (i.e., endometrial biopsy with a camera)					
D&C (Dilation and curettage)					
Blood Tests (Pregnancy or hormone panel test)					

56. **[IF "TEST RECEIVED" IS SELECTED]** How would you rate the <u>discomfort you experienced after</u> the following tests? Please rate the discomfort of the following tests, with 1 being no discomfort and 5 being high discomfort.

[LINK TO DESCRIPTIONS]

**[DISPLAY ONLY TESTS RECEIVED]**	No discomfort (1)	(2)	Moderate discomfort (3)	(4)	High discomfort (5)
Pelvic exam	**[RADIO BUTTONS]**				
Transvaginal ultrasound					
Saline infusion sonohysterogram (i.e., transvaginal ultrasound with saline)					
Endometrial biopsy					
Hysteroscopy guided biopsy (i.e., endometrial biopsy with a camera)					
D&C (Dilation and curettage)					
Blood Tests (Pregnancy or hormone panel test)					

57. **[IF “TEST RECEIVED” IS SELECTED]** How long did it take to complete all testing and receive a diagnosis/begin treatment for your abnormal vaginal bleeding? Please consider from the day you first discussed your symptoms with your doctor.

   a. Less than 1 month

   b. 1 to 3 months

   c. 4 to 6 months

   d. 7 to 12 months

   e. 1 to 2 years

   f. More than 2 years

   g. Testing for abnormal vaginal bleeding has not been completed

58. [IF “TEST RECEIVED” IS SELECTED] How much did the following factors of testing impact your quality of life? Please answer if you agree or disagree with the following statements.

	Strongly disagree (1)	Somewhat disagree (2)	Neutral (3)	Somewhat agree (4)	Strongly agree (5)
Testing took up a lot of my time	[RADIO BUTTONS]				
Testing interfered with my daily activities (e.g., going to work)					
Waiting to have tests conducted caused me anxiety and stress					
Waiting for results for my tests caused me anxiety and stress					

59. **[IF "NO TEST RECEIVED" OR "SOME TESTS RECEIVED" IS SELECTED]** Why did you decide not to get all tests your doctor recommended for abnormal bleeding? Please rate the influence of the following factors, with 1 being not influential at all and 5 being extremely influential

	Not influential at all (1)	Somewhat influential (2)	Slightly influential (3)	Very influential (4)	Extremely influential (5)
It was inconvenient traveling to the test	**[RADIO BUTTONS]**				
It was inconvenient taking time out of my day to get the test					
I was afraid of getting a bad result					
I was afraid the test would hurt					
I disliked how invasive the test would be					
The doctor performing the tests was not my preferred gender doctor					
It was too expensive					
**[IF "NO TEST RECEIVED]** Doctor recommended I did not get tested					

**[IF SOME TESTS RECEIVED]** Doctor said I did not need all tests	
The abnormal vaginal bleeding stopped	
I have a history of assault and am uncomfortable with gynecological tests	

60. **[IF “NO TEST RECEIVED” IS SELECTED]** What would motivate you to get tested for abnormal vaginal bleeding? Please rate the following factors, with 1 being not motivating at all and 5 being extremely motivating.

	Not motivating at all (1)	Slightly motivating (2)	Somewhat motivating (3)	Very motivating (4)	Extremely motivating (5)
There is more blood when I have abnormal vaginal bleeding	**[RADIO BUTTONS]**				
Abnormal vaginal bleeding happens more frequently					
I experience other symptoms that I think are related to my reproductive organs (e.g., cramps)					
I learn that abnormal bleeding is a serious problem					
There are new affordable testing options					
There are new more convenient testing options					
There are tests I can do by myself (i.e., self-testing like COVID-19)					
There are less invasive testing options					

My doctor recommends I get tested	

61. **[IF “NO TEST RECEIVED” AND “NOT ALL TESTS DISCUSSED” IS SELECTED AND “DOCTOR RECOMMENED NO TEST” IS NOT SELECTED]** If your doctor discussed the other forms of testing for abnormal vaginal bleeding with you, would you have agreed to receive one of them? Please select all you would be interested in receiving.

[LINK TO DESCRIPTIONS]

[DISPLAY TESTS DOCTOR DID NOT DISCUSS]

a. Pelvic exam
b. Transvaginal ultrasound
c. Saline infusion sonohysterogram (i.e., transvaginal ultrasound with saline)
d. Endometrial biopsy
e. Hysteroscopy guided biopsy (i.e., endometrial biopsy with a camera)
f. Dilation and curettage
g. Pregnancy blood test
h. Hormone panel blood test
i. No, I would still refuse testing

[SELECT ALL THAT APPLY]

62. **[IF "NOT SEEN HCP FOR ABNORMAL BLEEDING" IS SELECTED]** What tests for determining the cause of abnormal vaginal bleeding are you familiar with? Please select all that apply.

a. Pelvic exam
b. Transvaginal ultrasound
c. Saline infusion sonohysterogram (i.e., transvaginal ultrasound with saline)
d. Endometrial biopsy
e. Hysteroscopy guided biopsy (i.e., endometrial biopsy with a camera)
f. D&C (Dilation and curettage)
g. Pregnancy blood test
h. Hormone panel blood test
i. None of the testing methods

[SELECT ALL THAT APPLY]

63. **[IF “FAMILIAR WITH TESTS” IS SELECTED]** How did you learn about these tests? Please select all that apply.

   a. Conversation with healthcare provider
   b. Brochure from doctors’ office
   c. Message from pharmacy (e.g., brochure, email, mail)
   d. Message from insurance company (e.g., brochure, email, mail)
   e. Online research
   f. Radio or TV
   g. Conversations with friends and/or family
   h. Social media ads
   i. Other (please specify): **[FORCE TEXT]**

[SELECT ALL THAT APPLY]

64. How concerned were you about your abnormal vaginal bleeding at each of the following stages?

Please rate your concern regarding your abnormal vaginal bleeding at the following stages, with 1 being not concerned at all and 5 being extremely concerned.

	Not at all concerned (1)	Slightly concerned (2)	Somewhat concerned (3)	Very concerned (4)	Extremely concerned (5)
When the bleeding first started	[RADIO BUTTONS]				
When you noticed the bleeding wasn't normal					
[IF SEEN HCP IS SELECTED] When you decided to discuss the bleeding with your doctor					
[IF TESTING WAS DISCUSSED] After you discussed testing that will be done for the bleeding					
[IF TESTING WAS CONDUCTED] After you completed the testing that was done for the bleeding					
[IF TESTING WAS CONDUCTED] After you received the test results for your bleeding					
Today					
