# Supplementary material for: Barriers to the recognition and evaluation of abnormal uterine bleeding among individuals at increased risk for endometrial Cancer
Source: Gynecol Oncol Rep. 2026 Sep 24;67:102214. doi: 10.1016/j.gore.2026.102214 (PMC13636334; doi:10.1016/j.gore.2026.102214)
Supplement: Supplementary file 2 [file mmc2.docx]

| **Outcomes** | **Provider Type**, **n/N (%)** | | | | | | | |
| --- | --- | --- | --- | --- | --- | --- | --- | --- |
|  | Gynecologist | Primary Care Provider | Urgent Care/ Walk-in Clinic | Community Clinic | ER or Hospital | Telehealth | Retail Clinic | Other |
| Overall Distribution among participants who saw an HCP for bleeding (N=450) | 262/450  (58.2%) | 121/450  (26.9%) | 21/450  (4.7%) | 14/450  (3.1%) | 14/450  (3.1%) | 5/450  (1.1%) | 2/450  (0.4%) | 11/450 (2.4%) |
| Provider was not concerned about participant’s AUB | 67/262  (25.6%) | 44/121  (36.4%) | 6/21  (28.6%) | 1/14  (7.1%) | 0/14  (0%) | 0/5  (0%) | 1/2  (50%) | 6/11 (54.6%) |
| Underwent Diagnostic Evaluation | 231/262  (88.2%) | 101/121  (83.5%) | 16/21  (76.2%) | 12/14  (85.7%) | 13/14  (92.9%) | 5/5  (100%) | 2/2  (100%) | 7/11 (63.6%) |

Note: Only participants who reported seeing HCP for bleeding were included.
