# Supplementary material for: Barriers to the recognition and evaluation of abnormal uterine bleeding among individuals at increased risk for endometrial Cancer
Source: Gynecol Oncol Rep. 2026 Sep 24;67:102214. doi: 10.1016/j.gore.2026.102214 (PMC13636334; doi:10.1016/j.gore.2026.102214)
Supplement: Supplementary file 3 [file mmc3.docx]

| **Category/Question** | **PMB**  **(N=172)** | **No PMB (N=431)** | **P-value** |
| --- | --- | --- | --- |
| Time until AUB recognition  1-6 months  Greater than 6 months | 147 (85.5%)  25 (14.5%) | 327 (75.9%)  104 (24.1%) | 0.0112 |
| Recognition of AUB as Symptom of Cancer [1] | 19 (11.1%) | 34 (7.9%) | 0.0529 |
| Familiarity with Endometrial Biopsy  Yes | 94 (54.7%) | 181 (42.0%) | 0.0051 |
| Did the participant seek care?  No  Yes | 44 (25.6%)  128 (74.4%) | 109 (25.3%)  322 (74.7%) | 1.0000 |
| Provider not concerned about participant’s bleeding | 26 (15.1%) | 99 (23.0%) | 0.0345 |

[1] Only participants who reported being concerned about their bleeding were asked this question
